# *Epimeria abyssalis* sp. n. from the Kuril-Kamchatka Trench (Crustacea, Amphipoda, Epimeriidae)

**DOI:** 10.3897/zookeys.638.10329

**Published:** 2016-12-08

**Authors:** Michitaka Shimomura, Ko Tomikawa

**Affiliations:** 1 Kitakyushu Museum of Natural History and Human History, 2-4-1 Higashida, Yahatahigashi-ku, Kitakyushu 805-0071, Japan; 2 Graduate School of Education, Hiroshima University, 1-1-1 Kagamiyama, Higashi-Hiroshima 739-8524, Japan

**Keywords:** Epimeria, Epimeriidae, Kuril-Kamchatka Trench

## Abstract

A new deep-sea epimeriid, *Epimeria abyssalis* is described from the Kuril-Kamchatka Trench, in the northwestern Pacific. This species differs from its congeners in having a short rostrum and a telson with deep and narrow Y-shaped excavation. *Epimeria abyssalis* is the deepest recorded *Epimeria* species. A key to the north Pacific species of *Epimeria* is provided.

## Introduction

*Epimeria* Costa, 1851, is the largest genus of the family Epimeriidae Boeck, 1871 and includes 54 species (WoRMS 2016), which it is nearly cosmopolitan and was previously recorded between 0 and 3710 m depth. Among these, seven species have to date been reported from the North Pacific: *Epimeria cora* J. L. Barnard, 1971 at 2086 m, off Oregon (Barnard 1971), *Epimeria morronei* Winfield et al., 2012 at 1395–2093 m, Gulf of California and off the west coast of Baja, Mexico (Winfield et al. 2012; Hendrickx et al. 2014), *Epimeria ortizi* Varela & García-Gómez, 2015 at 198–1224 m, Gulf of Mexico (Varela and García-Gómez 2015), *Epimeria pacifica* Gurjanova, 1955 at 1430–1450 m, the Japan Trench (Gurjanova 1955), *Epimeria pelagica* Birstein & Vinogradov, 1958, caught in a plankton net sampling at 0–8000 m, the Kuril-Kamchatka Trench and the Japan Trench (Birstein and Vinogradov 1958; Nagata 1963), *Epimeria subcarinata* Nagata, 1963 at 2230 m, off Onagawa, the northwestern Pacific (Nagata 1963), and *Epimeria yaquinae* McCain, 1971 at 2800–2862 m, off Oregon (McCain 1971).

This deep-sea survey yielded an undescribed species *Epimeria* from an abyssal zone of the Kuril-Kamchatka Trench, the northwestern Pacific, which is described and illustrated in this work.

## Materials and methods

Amphipod specimens were collected during a survey of deep-sea benthic fauna of northern Japan by the R/V “Hakuho-Maru” of the Ocean Research Institute, University of Tokyo in 2001 (now the ship belongs to Japan Agency for Marine-Earth Science and Technology), from station KH-01-02-XR-8 and XR-12. The gear used for the collection was an ORE beam trawl of 4 m span (mesh size approx. 5 mm). Samples were elutriated on board through a 0.5 mm mesh sieve. The specimens retained were fixed and preserved in 70% ethanol. Appendages of each individual were dissected and observed using a compound and stereo microscopes. Total length was measured from the tip of the head to the end of the telson. Terminology follows Coleman (2007). The type specimens are deposited in the Kitakyushu Museum of Natural History and Human History, Japan (KMNH).

## Systematics

### Epimeria

Taxon classificationAnimaliaAmphipodaEpimeriidae

Costa in Hope, 1851

#### Type-species.

*Epimeria tricristata* Costa in Hope, 1851 (= *Gammarus corniger* Fabricius, 1779)

### Epimeria abyssalis sp. n.

Taxon classificationAnimaliaAmphipodaEpimeriidae

http://zoobank.org/1A3E4D57-208C-40F9-8B63-484F2304A8B2

Figures 1 , 2 , 3 , 4 , 5 , 6 , 7 , 8 , 9 , 10 , 11

#### Material examined.

***Holotype*.** Ovigerous ♀ (53 mm) (KMNH IvR 500905), with 5 eggs, Sta. KH-01-02-XR-12, 41°37.67N, 146°54.19E–41°26.20N, 146°23.03E, 5473–5484 m depth, muddy bottom, Kuril–Kamchatka Trench, 22–23 September 2001, 4 m ORE beam trawl, towed by R/V “Hakuho-Maru”.

***Paratypes*.** 1 ovigerous ♀ (47 mm) (KMNH IvR 500906), 1 juvenile ♀ (22 mm) (KMNH IvR 500907), Sta. KH-01-02-XR-8, 41°50.08 N 145°37.85E–41°49.70N 145°35.18E, 5695–5664 m depth, muddy bottom, Kuril–Kamchatka Trench, 19 September 2001, 4 m ORE beam trawl, towed by R/V “Hakuho-Maru”.

#### Description of the holotype.

**Figure 1.**
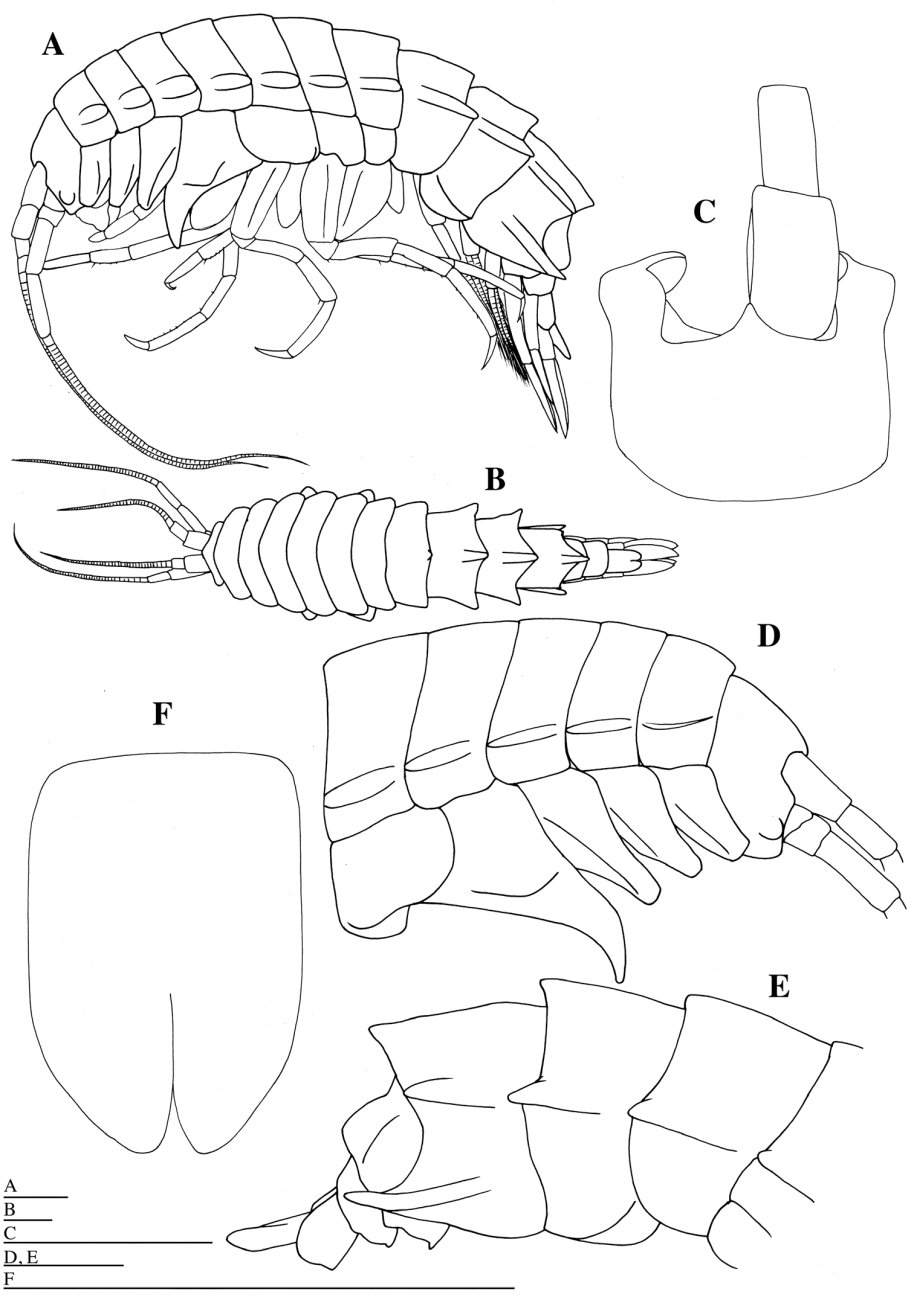
*Epimeria abyssalis* sp. n., holotype female: **A** habitus, lateral **B** habitus, dorsal **C** head and articles 1 and 2 of right antenna 1, dorsal **D** anterior part of body, lateral **E** posterior part of body, lateral **F** telson, dorsal. Scale bars: 5 mm.

Rostrum (Fig. 1A, D, C) short, 0.2 times as long as head, not reaching one third of first article of antenna 1. Head (Fig. 1D) ventral lobe blunt. No eye pigments but swelling present in expected eye position. Pereonites 1–7 (Fig. 1A, B, D) without dorsal carinae: pereonite 1 0.8 times as long as head (excluding rostrum); pereonite 2 0.9 times as long as pereonite 1; pereonites 1–7 each with short dorsolateral processes, lacking mid-dorsal processes; pereonite 7 with short dorsolateral and mid-dorsal process. Pleonites 1–3 (Fig. 1A, B, E) with dorsal carinae and posterolateral processes: dorsal carinae of pleonites 1 and 2 not reaching apex of posterolateral processes; dorsal carina of pleonite 3 reaching apex of posterolateral processes. Epimeral plate 1 (Fig. 1A, E) with rounded posteroventral angle; epimeral plate 2 (Fig. 1A, E) with less rounded posteroventral angle; epimeral plate 3 (Fig. 1A, E) with posteroventral angle produced into a large tooth, reaching apex of dorsal carina of pleonite 3.

*Urosomites* 1–3 (Fig. 1A, E) without dorsal processes, extremely low rounded lobe on urosomite 1: urosomite 1 longest; urosomite 2 shortest, 0.4 times as long as urosomite 1; urosomite 3 1.7 times as long as urosomite 2.

**Figure 2.**
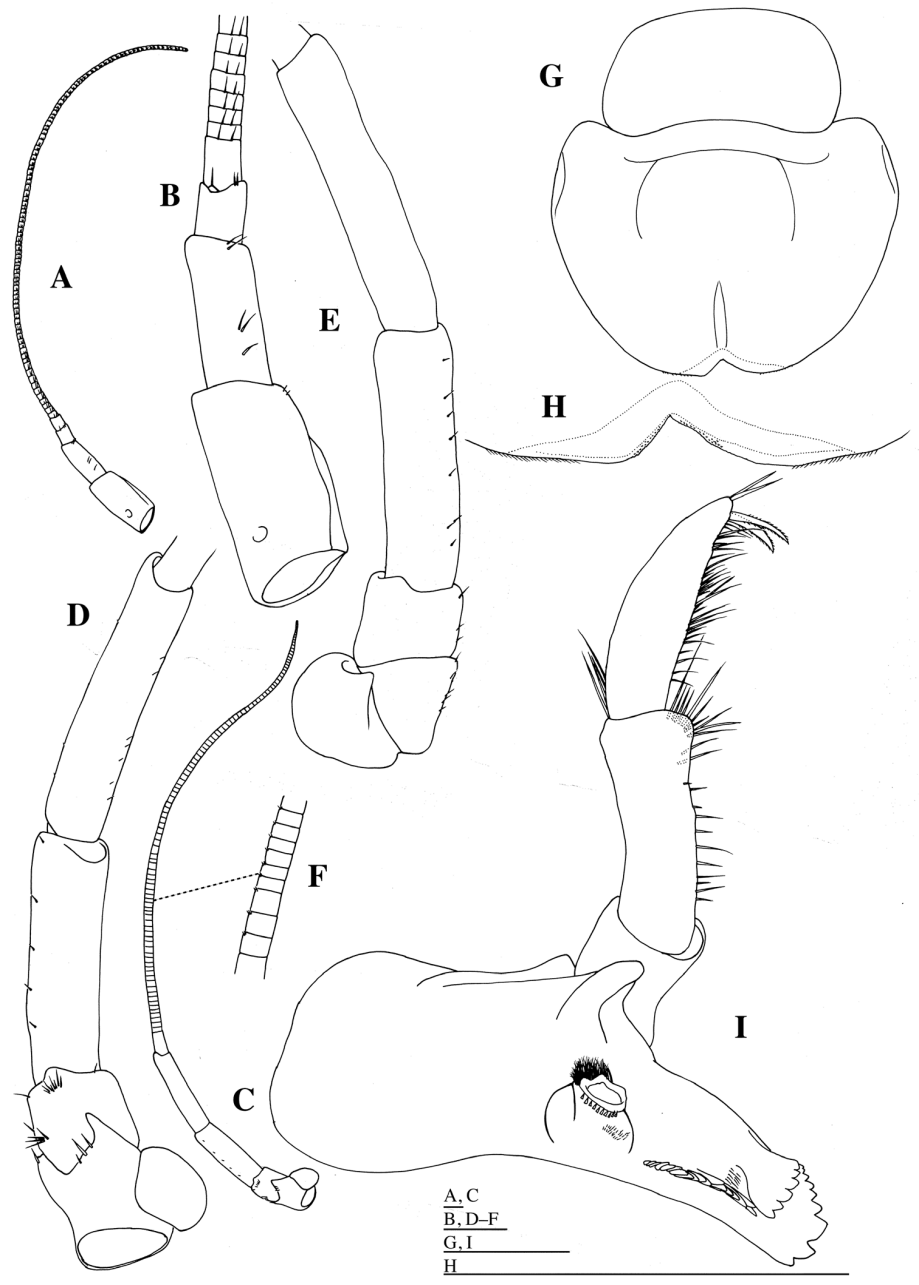
*Epimeria abyssalis* sp. n., holotype female: **A** left antenna 1, medial **B** basal part of left antenna 1, medial **C** left antenna 2, medial **D** basal part of left antenna 2, medial **E** basal part of left antenna 2, lateral **F** flagella of left antenna 2, medial **G** labium, dorsal **H** anterior part of labium, dorsal **I** left mandible, medial. Scale bars: 1 mm.

*Antenna 1* (Fig. 2A, B) peduncle without teeth, length of articles 1:2:3 approximately 5:3:1; article 1 twice as long as width; accessory flagellum 1-articulate, scale-like; primary flagellum of 102 articles. Antenna 2 (Fig. 2C–F): article 1 mediodistally projected; article 2 distolaterally projected; article 3 bluntly projected distolaterally; article 4 0.8 times as long as article 5; article 5 longest; flagellum of 104 articles.

**Figure 3.**
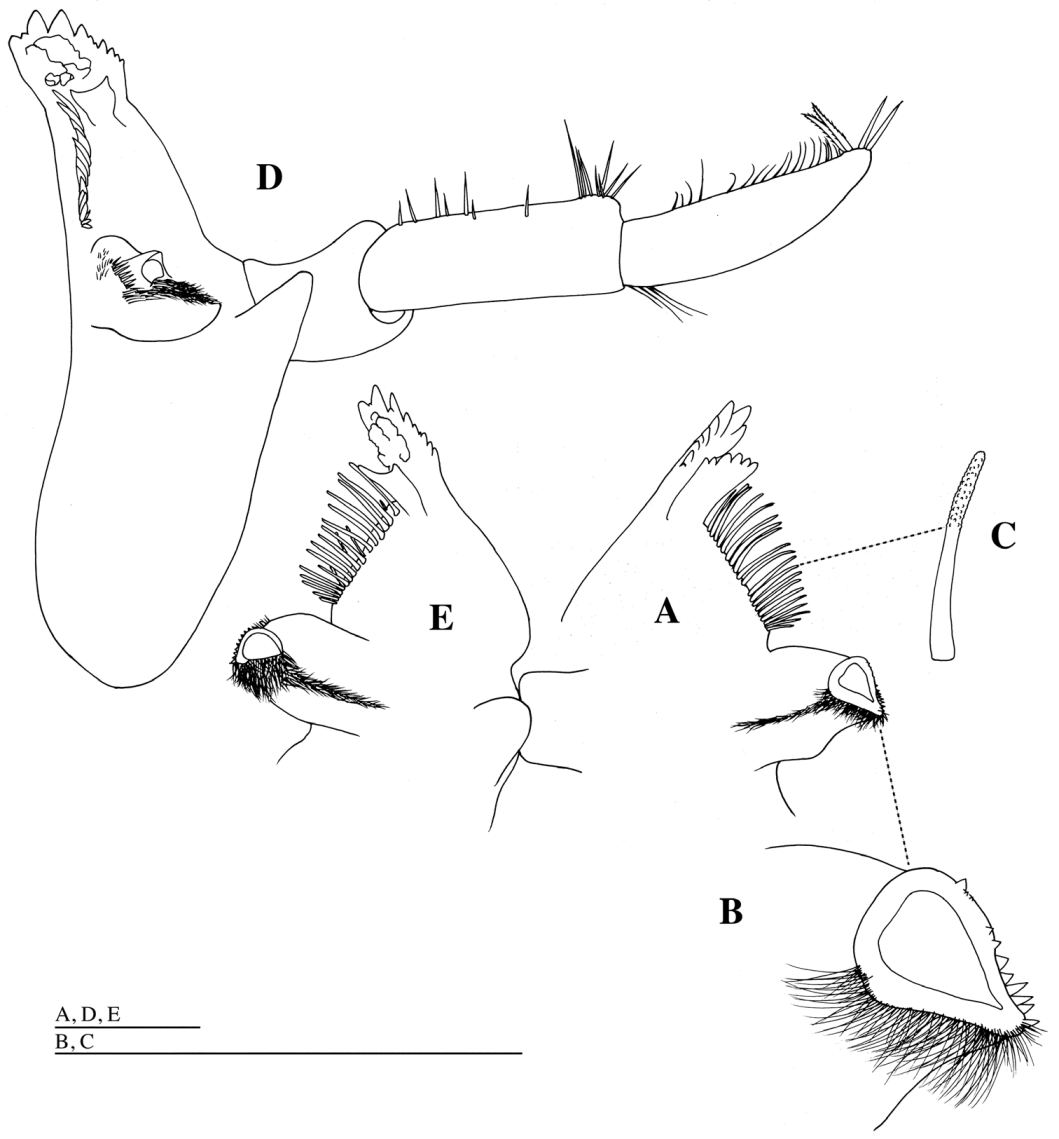
*Epimeria abyssalis* sp. n., holotype female: **A** molar process, incisor, lacinia mobilis and setal row of left mandible, dorsal **B** molar process of left mandible, dorsal **C** seta of setal row of left mandible, dorsal **D** right mandible, medial **E** molar process, incisor and setal row of right mandible, dorsal. Scale bars: 1 mm.

**Figure 4.**
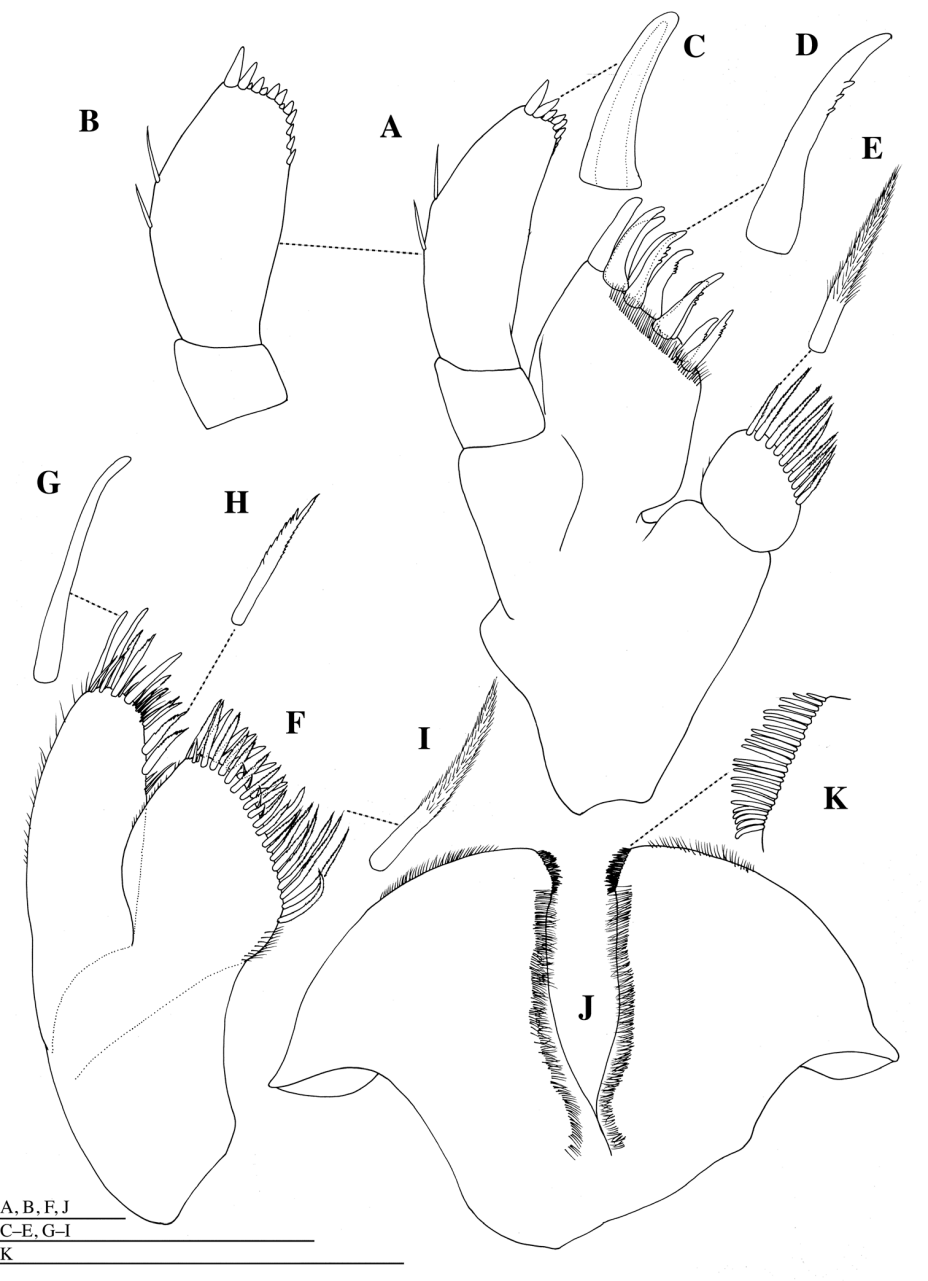
*Epimeria abyssalis* sp. n., holotype female: **A** left maxilla 1, dorsal **B** articles 1 and 2 of palp of left maxilla 1, dorsal **C** seta on article 2 of palp of left maxilla 1, dorsal **D** seta on outer plate of left maxilla 1, dorsal **E** seta on inner plate of left maxilla 1, dorsal **F** left maxilla 2, dorsal **G** seta on outer plate of left maxilla 2, dorsal **H** seta on outer plate of left maxilla 2, dorsal **I** seta on inner plate of left maxilla 2, dorsal **J** lower lip, dorsal. Scale bars: 1 mm.

**Figure 5.**
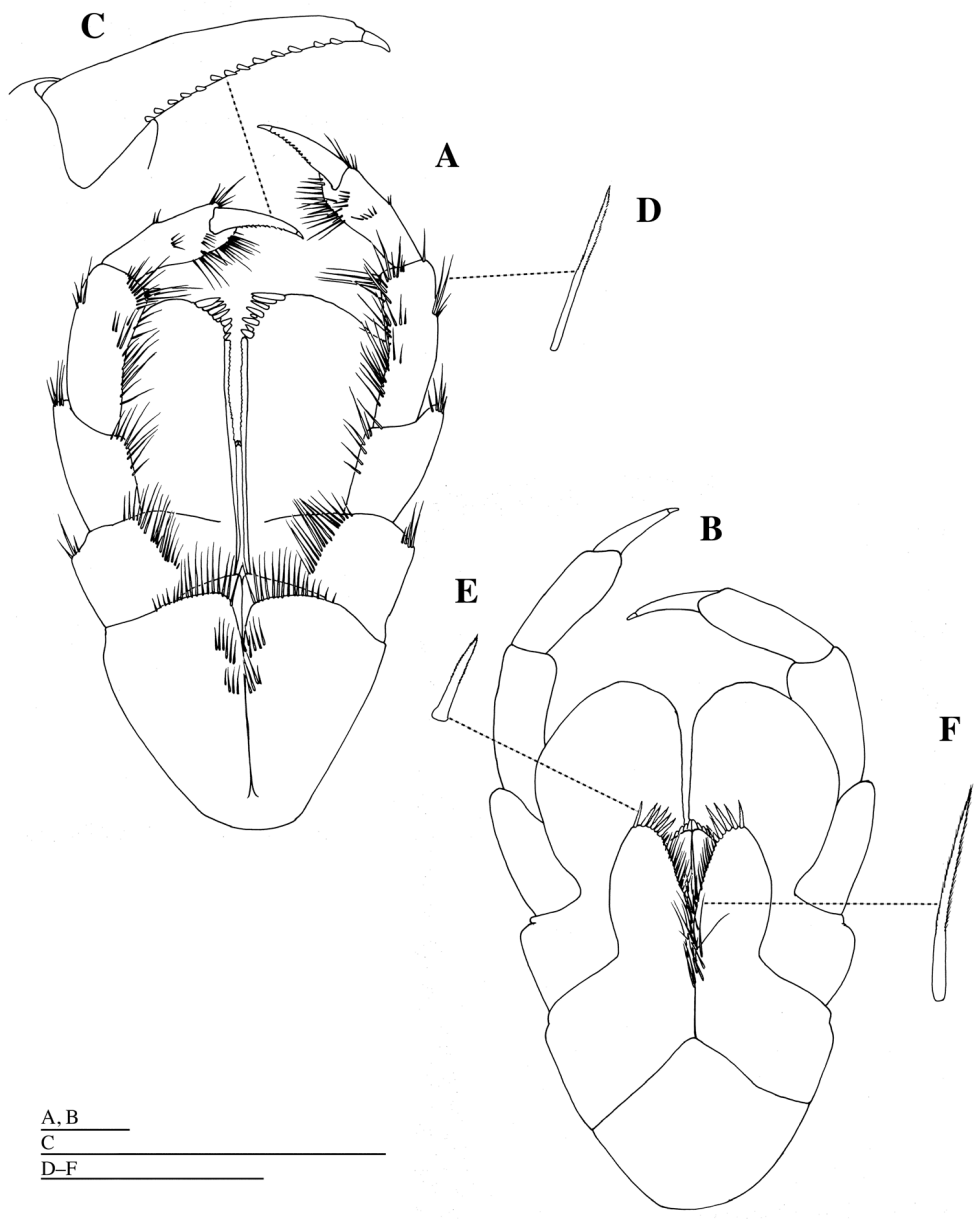
*Epimeria abyssalis* sp. n., holotype female: **A** left maxilliped, ventral **B** left maxilliped, dorsal (omitted setae excluding setae on inner plates), dorsal **C** article 4 of left maxillipedal palp, ventral **D** seta on article 2 of left maxillipedal palp, ventral **E** seta on inner plate of left maxilliped, dorsal **F** seta on inner plate of left maxilliped, dorsal. Scale bars: 1 mm.

*Labrum* (= upper lip) (Fig. 2G, H) with shallow notch distally; epistome broadly rounded. Mandible (Figs 2I, 3A–E): incisor and lacinia mobilis strongly dentate, left incisor and lacinia mobilis 9- and 6-dentate, respectively; molar produced and triturative, densely setose medially, with acute teeth distally; mandibular palp (Figs 2I, 3D) long; article 1 shortest; article 2 as long as article 3, sparsely setose medially; article 3 with some simple setae medially, two setulate and two simple long setae apically. Maxilla 1 (Fig. 4A–E): inner plate ovate, with ten stout plumose setae distally; outer plate distal margin oblique, with ten weakly serrate or unarmed robust setae; palp exceeding outer plate; palp article 1 short; palp article 2 2.9 times as long as article 1, with two simple setae laterally, and with stout setae distally and medially. Maxilla 2 (Fig. 4F–I): inner plate with stout plumose setae distally, and with short simple setae medially and laterally; outer plate stout with simple short setae laterally, and with simple and crenulate setae distally. Maxilliped (Fig. 5A–F): inner plate moderately narrow, with long plumose setae medially and short plumose setae distally; outer plate broadly rounded distally, reaching two thirds the length of second article of maxillipedal palp; palp articles 1 and 2 with plumose setae distolaterally and medially; article 3 with row of short, stout setae medially and short claw apically. Lower lip (= labium) (Fig. 4J, K) with stout setae distomedially, fine setae medially and distolaterally; broad hypopharyngeal lobes; lateral processes narrow; inner lobe absent.

**Figure 6.**
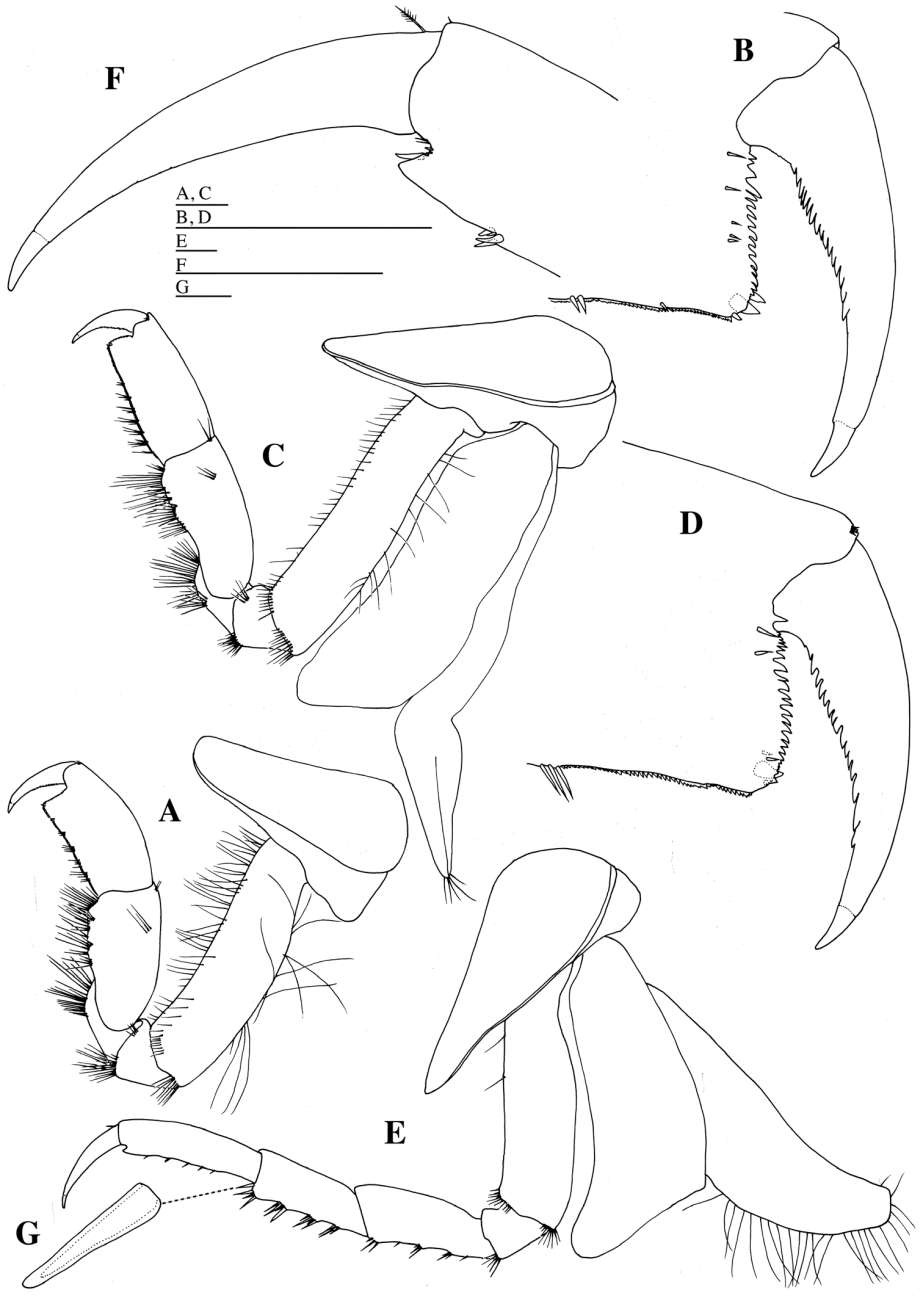
*Epimeria abyssalis* sp. n., holotype female: **A** left pereopod 1, lateral **B** distal part of propodus and dactylus of left pereopod 1, medial **C** left pereopod 2, lateral **D** distal part of propodus and dactylus of left pereopod 2, medial **E** left pereopod 3, lateral **F** distal part of left pereopod 3, lateral **G** seta on carpus of left pereopod 3, lateral. Scale bars: 1 mm.

*Gnathopod 1* (Figs 1A, D, 6A, B): coxa slender, with blunt apex; anterior margin of coxa slightly concave; basis longest, with numerous fine setae anteriorly and posteriorly, and with groups of setae anterodistally and posterodistally; ischium triangular, with many long setae distally; merus slightly longer than ischium, with many long setae distally; carpus 0.6 times as long as basis, with groups of long setae posteriorly; propodus stout, as long as carpus, crenulate posteriorly, with groups of short setae on posterior border, and with two robust and some slender setae distally; posterodistal angle squared; palmar margin transverse strongly serrate; dactylus slender, slightly curved, serrate posteriorly, with acute unguis apically.

*Gnathopod 2* (Figs 1A, D, 6C, D): coxa as wide as coxa 1, with blunt apex; anterior margin of coxa slightly concave; basis longest, slender than basis of gnathopod 1, with numerous fine setae anteriorly and posteriorly, and with groups of setae anterodistally and posterodistally; ischium trapezoidal, with many long setae distally; merus slightly longer than ischium, with many long setae distally; carpus half as long as basis, with groups of long setae posteriorly; propodus stout, slightly narrower than propodus of gnathopod 1, 0.9 times as long as carpus, crenulate posteriorly, with groups of short setae posteriorly, and with 1 robust and some slender setae distally; posterodistal angle squared; palmar margin transverse, strongly serrate; dactylus slender, slightly curved, serrate posteriorly, with acute unguis apically.

*Pereopod 3* (Figs 1A, D, 6E, G): coxa as wide as coxa 2, with blunt apex; anterior margin of coxa slightly concave; basis 0.9 times as long as basis of gnathopod 2, sparsely setose anteriorly, and with groups of setae anterodistally and posteodistally; ischium trapezoidal, with some setae distally; merus 2.9 times as long as width, 2.8 times as long as ischium, with groups of setae posteriorly; carpus 0.9 times as long as merus, with groups of setae posteriorly; propodus 1.3 times as long as carpus, acutely projected posterodistally, with groups of short setae posteriorly; dactylus slender, slightly curved, 0.7 times as long as propodus, lacking serration, with acute unguis apically.

**Figure 7.**
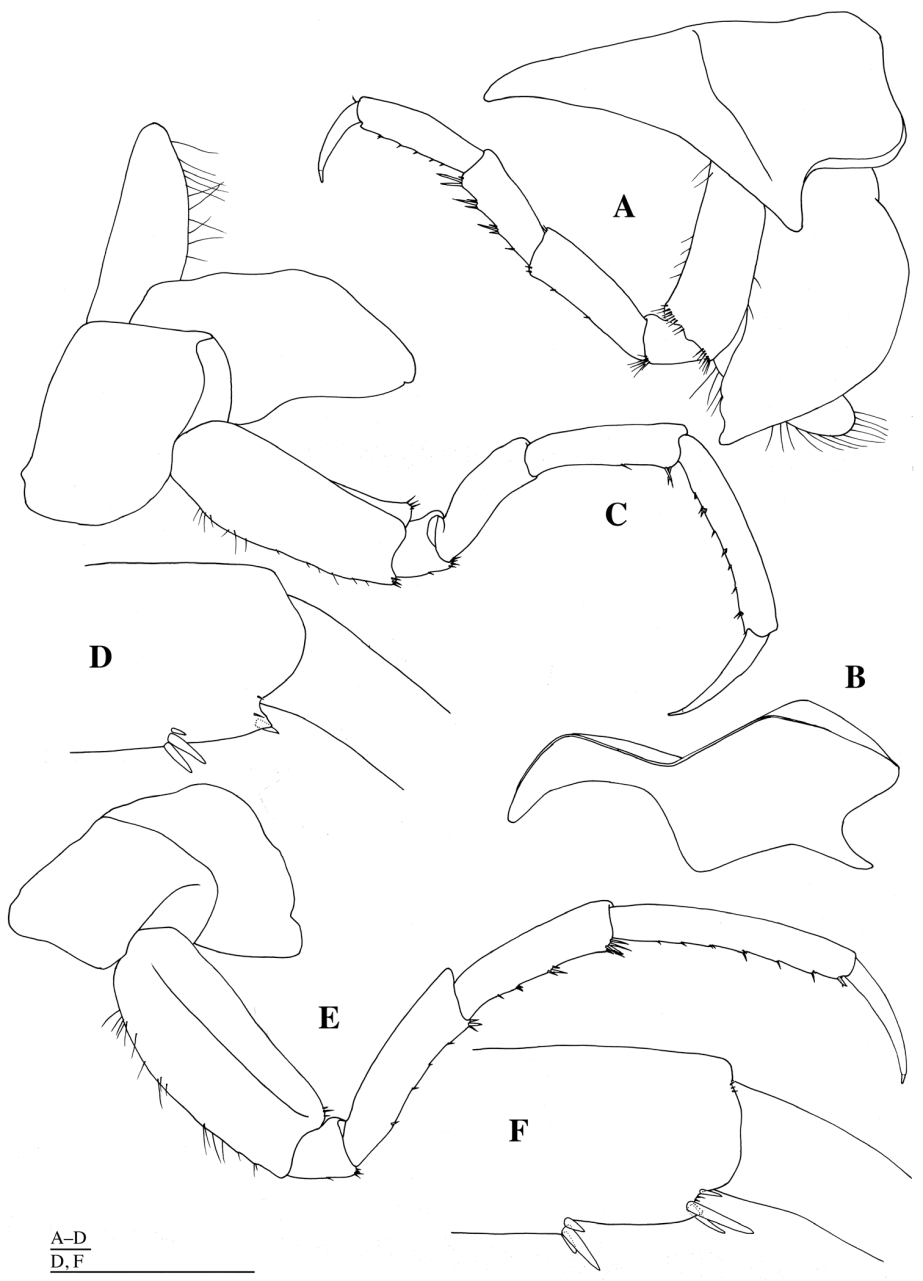
*Epimeria abyssalis* sp. n., holotype female: **A** left pereopod 4, lateral **B** coxa of left pereopod 4, dorsal **C** left pereopod 5, lateral **D** distal part of propodus of left pereopod 5, lateral **E** left pereopod 6, lateral **F** distal part of propodus of left pereopod 6, lateral. Scale bars: 1 mm.

*Pereopod 4* (Figs 1A, D, 7A, B): coxa 1.9 times as wide as coxa 3, produced into posterodistal cusp directed posterodistally, laterally projected at mid part; anterior margin of coxa slightly concave; basis as long as basis of pereopod 3, sparsely setose anteriorly and posteriorly, and with groups of setae anterodistally and posterodistally; ischium trapezoidal, with some setae distally; merus 3.6 times as long as width, 2.8 times as long as ischium, sparsely setose posteriorly; carpus 0.7 times as long as merus, with groups of long setae posteriorly; propodus 1.2 times as long as carpus, acutely projected posterodistally, with groups of short setae posteriorly; dactylus slender, slightly curved, 0.7 times as long as propodus, lacking serration, with acute unguis apically.

*Pereopod 5* (Figs 1A, D, 7C, D): coxa as wide as coxa 4, subrectangular, without anterodistal and posterodistal projections; anterior margin of coxa broadly rounded; basis as long as basis of pereopod 4, 1.5 times as wide as basis of pereopod 4, setose anteriorly, and with groups of setae anterodistally and posterodistally; ischium trapezoidal, with some setae distally; merus 3.3 times as long as width, 2.9 times as long as ischium; carpus 1.3 times as long as merus, sparsely setose anteriorly; propodus 1.4 times as long as carpus, acutely projected posterodistally, with groups of short setae anteriorly; dactylus very long, slender, slightly curved, 0.6 times as long as propodus, lacking serration, with acute unguis apically.

*Pereopod 6* (Figs 1A, 7E, F): coxa 0.6 times as wide as coxa 5, subrectangular, ventrally concave, without anterodistal and posterodistal projections; anterior margin of coxa nearly straight; basis ventrally convex, nearly straight dorsally, with longitudinal keel laterally, as long as basis of pereopod 5, 1.4 times as wide as basis of pereopod 5, setose anteriorly, and with groups of setae posterodistally; ischium trapezoidal, with some setae distally; merus 3.8 times as long as width, 3.6 times as long as ischium, with groups of short setae anteriorly; carpus 0.8 times as long as merus, with groups of setae anteriorly and anterordistally; propodus 1.4 times as long as carpus, without projection posterodistally, with groups of short setae anteriorly; dactylus very long, slender, slightly curved, 0.5 times as long as propodus, lacking serration, with acute unguis apically.

**Figure 8.**
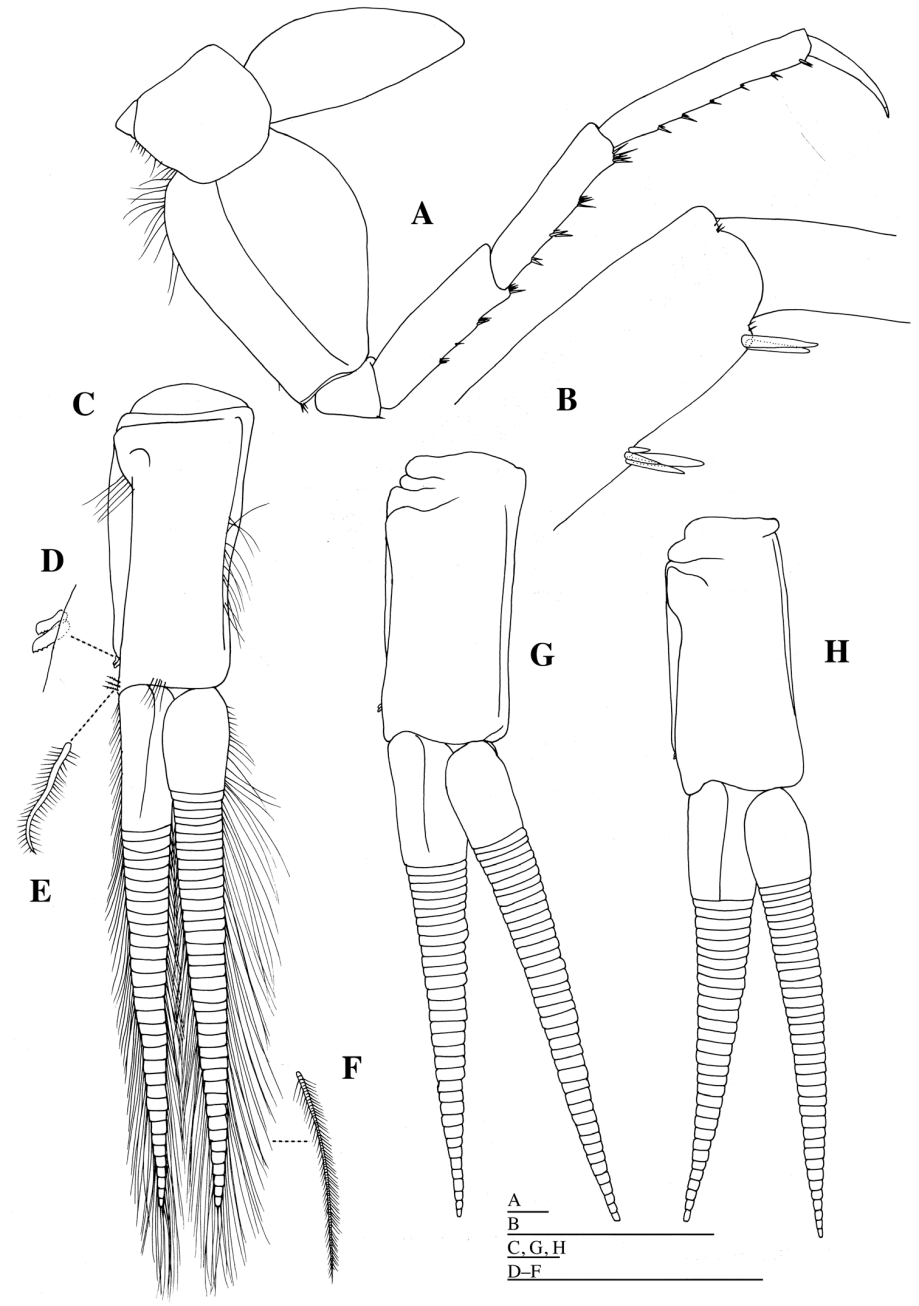
*Epimeria abyssalis* sp. n., holotype female: **A** left pereopod 7, lateral **B** distal part of propodus of left pereopod 7, lateral **C** left pleopod 1, dorsal **D** coupling hooks on peduncle of left pleopod 1, dorsal **E** seta on peduncle of left pleopod 1, dorsal **F** seta on outer ramus of left pleopod 1, dorsal. Scale bars: 1 mm.

*Pereopod 7* (Figs 1A, 8A, B): coxa 0.6 times as wide as coxa 5, subquadrate, ventrally convex, without anterodistal and posterodistal projections; anterior margin of coxa nearly straight; posteroventral corner of coxa very broadly rounded; basis broadest, convex ventrally and dorsally, 1.4 times as long as width, as long as basis of pereopod 6, 1.6 times as wide as basis of pereopod 6, setose anteriorly, and with groups of setae posterodistally; ischium trapezoidal, with some setae distally; merus 3.4 times as long as width, 2.8 times as long as ischium, with groups of short setae anteriorly; carpus as long as merus, with groups of setae anteriorly and anterordistally; propodus 1.3 times as long as carpus, without projection posterodistally, with groups of short setae anteriorly; dactylus very long, slender, slightly curved, half as long as propodus, lacking serration, with acute unguis apically.

*Coxal gills* on gnathopod 2 and pereopods 3–7 (Figs 6C, E, 7A, B, D, 8A). *Oostegites* (= brood plates) (Figs 6C, E, 7A, B) with numerous marginal setae; oostegites of gnathopod 2 and pereopod 3 longer than bases and coxal gills; oostegites of pereopod 4 longer than basis and shorter than coxal gill; oostegite of pereopod 5 as long as basis and shorter than gill.

*Pleopods 1–3* (Fig. 8C–H) similar in shape, decreasing in length posteriorly: peduncle broad, subrectangular, with many setae laterally, three plumose setae mediodistally and two coupling hooks (= retinacula); inner ramus as long as outer ramus; rami articulated with many plumose setae medially and laterally.

**Figure 9.**
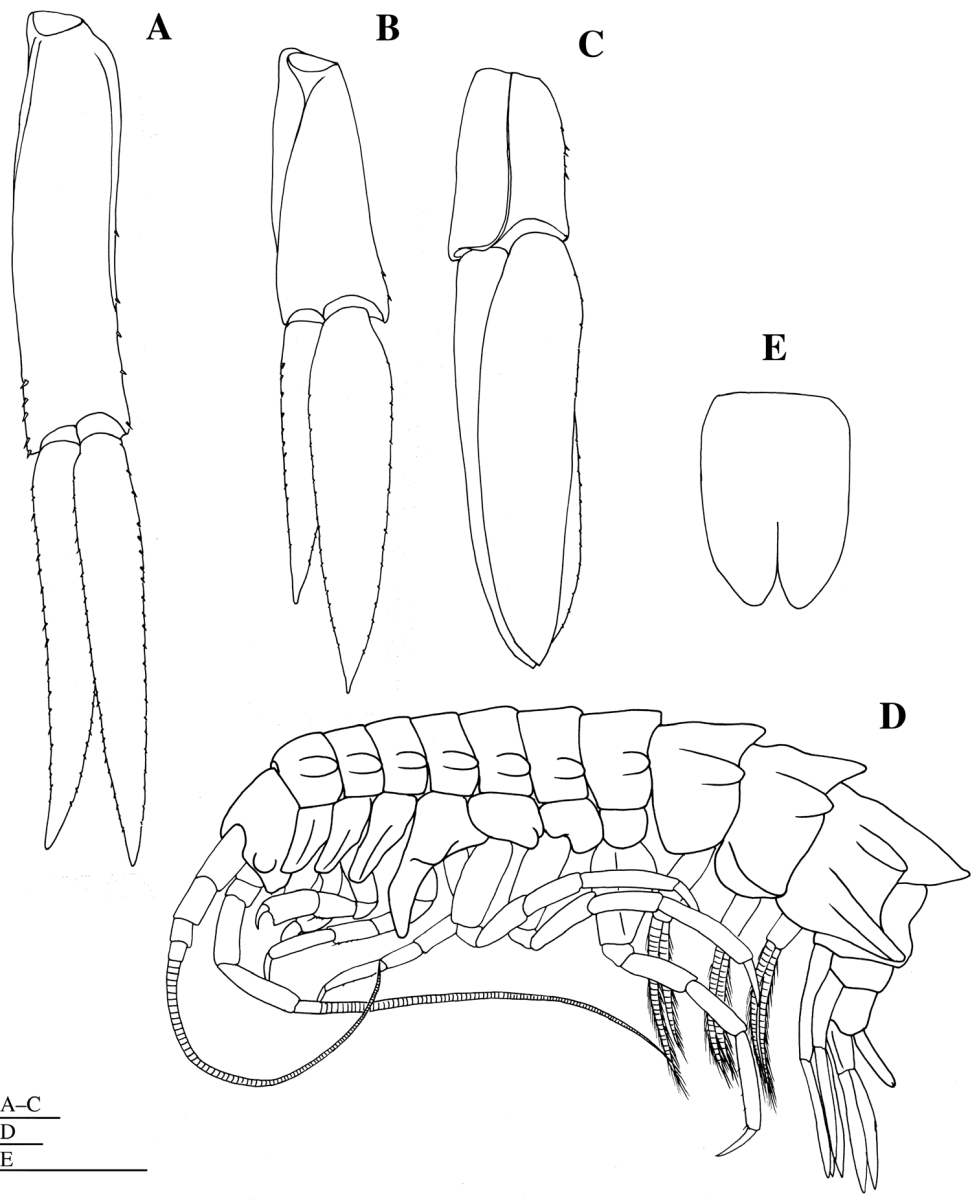
*Epimeria abyssalis* sp. n., **A–C** holotype female, **D, E** paratype female (KMNH IvR 500907): **A** left uropod 1, dorsal **B** left uropod 2, dorsal **C** left uropod 3, dorsal **D** habitus, lateral E telson, dorsal. Scale bars: 1 mm.

*Uropod 1* (Fig. 9A): peduncle subequal in length to inner ramus, with five short robust setae medially and five short robust setae laterally; inner ramus slightly curved medially, acutely pointed, with many short robust setae on margin; outer ramus as long as inner ramus, acutely pointed, with many short robust setae on margin.

*Uropod 2* (Fig. 9B) 0.8 times as long as uropod 1; peduncle subequal in length to inner ramus, increasing in width distally, with two short robust setae laterally; inner ramus acutely pointed, with many short robust setae on margin; outer ramus 1.4 times as long as inner ramus, acutely pointed, with many short robust setae on margin.

*Uropod 3* (Fig. 9C) 0.9 times as long as uropod 2; peduncle 0.4 times as long as inner ramus, increasing in width distally, with five short robust setae laterally; inner ramus broadest, moderately blunt apically, with sparse robust setae laterally; outer ramus as long as inner ramus, moderately blunt apically, with sparse robust setae laterally.

*Telson* (Fig. 1F) 1.5 times as long as wide, with deep and narrow Y-shaped excavation, without setae; distal cleft to 0.4 of total length of telson.

#### Description of the paratype female

**Figure 10.**
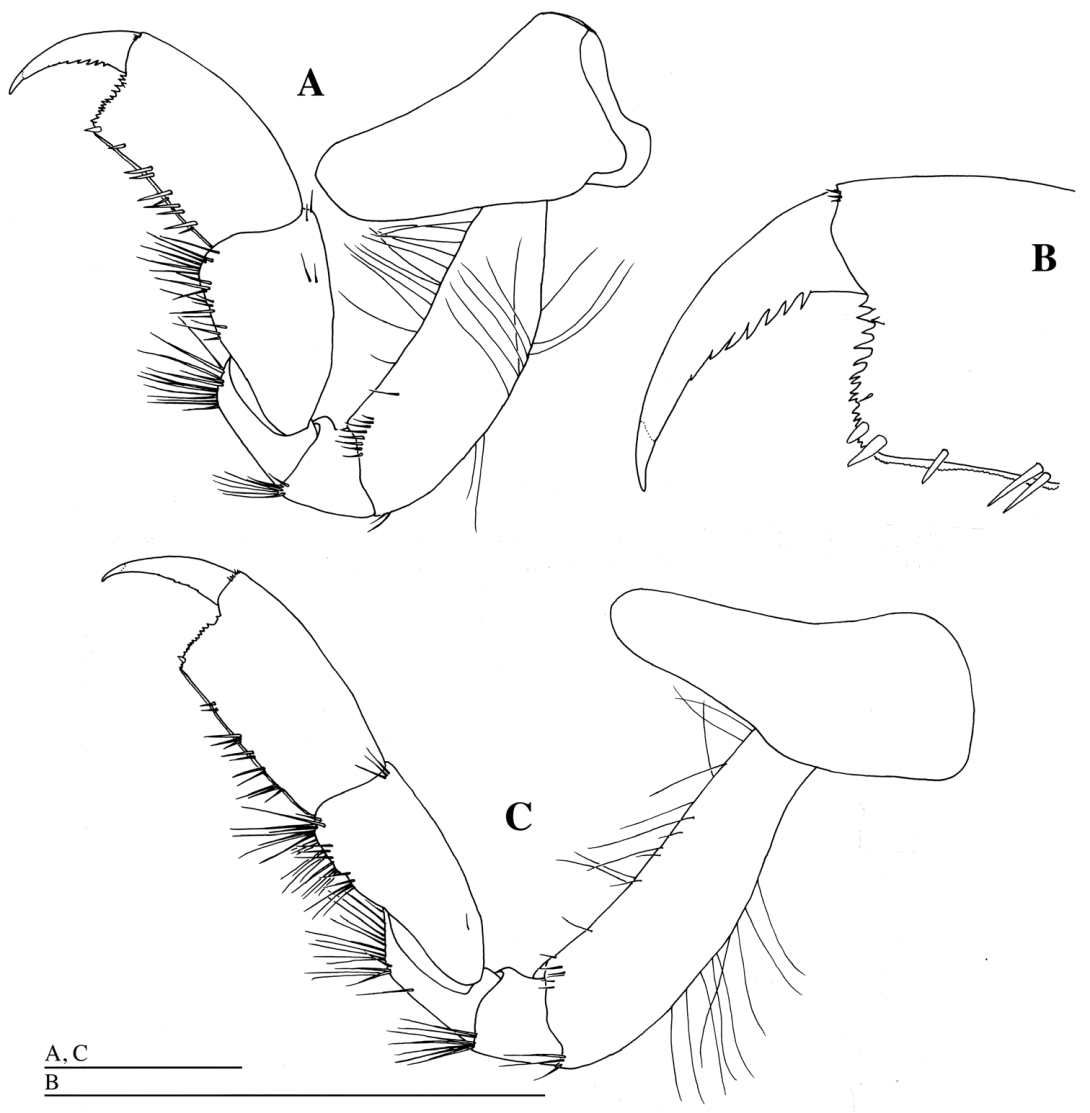
*Epimeria abyssalis* sp. n., paratype female (KMNH IvR 500907): **A** left pereopod 1, lateral **B** distal part of propodus and dactylus of left pereopod 1, lateral **C** left pereopod 2, lateral. Scale bars: 1 mm.

**(KMNH IvR 500907).** Similar to holotype in morphology of all appendages (Figs 9D, E, 10A–C). Pleonites 1–3 (Fig. 9D) with dorsal carinae and posterolateral processes; dorsal carinae of pleonites 1 and 2 reaching apex of posterolateral processes. Epimeral plate 3 (Fig. 9D) with pointed posteroventral angle, reaching apex of dorsal carina of pleonite 3.

*Telson* (Fig. 9E) 1.4 times as long as wide, with deep and narrow Y-shaped excavation, without setae.

#### Coloration.

**Figure 11.**
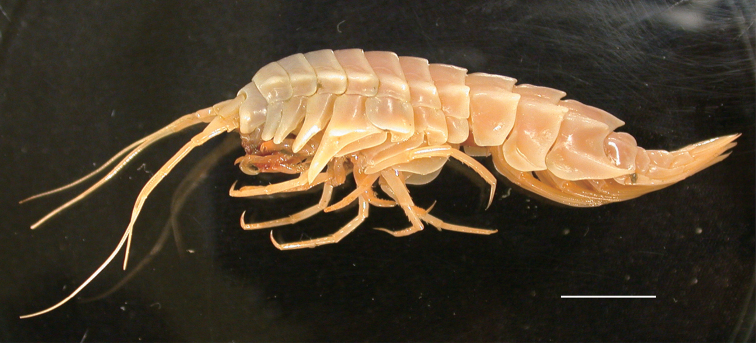
*Epimeria abyssalis* sp. n., paratype female (KMNH IvR 500906) photographed on board shortly after sampling. Scale bar: 10 mm.

Body (Fig. 11) and appendages excluding maxilliped cream-colored; distal part of maxilliped brownish red.

#### Remarks.

*Epimeria abyssalis* sp. n. can be identified and separated from other species of the genus by the following combination of characters: rostrum short, 0.2 times as long as head; eyes absent; pereonites 1–7 without dorsal carinae; palmar margins of propodi of gnathopods 1–2 transverse, strongly serrate; coxae 1–3 each with blunt apex; coxa 4 produced into posterodistal cusp directed posterodistally, laterally projected at mid part; anterior margin of coxa 4 slightly concave; coxa 5 as wide as coxa 4, subrectangular, without anterodistal and posterodistal projections; anterior margin of coxa 5 broadly rounded; basis of pereopod 7 broadest, as long as basis of pereopod 6, 1.6 times as wide as basis of pereopod 6; and telson 1.5 times as long as wide, with deep and narrow Y-shaped excavation, without setae.

*Epimeria abyssalis* sp. n. is close to *Epimeria pelagica* and *Epimeria yaquinae*, with which it shares a short rostrum, pereon without dorsal carinae, and coxa 5 lacking posterodistal projection are shared by *Epimeria pelagica* and *Epimeria yaquinae*. *Epimeria abyssalis* is distinguished from *Epimeria pelagica* by the following features (those of *Epimeria pelagica* in parentheses): eyes absent (present); article 1 of antenna 1 twice as long as wide (as long as wide); posterodistal angle of propodi of gnathopods 1 and 2 nearly right angle squared, (obtuse angle); coxa 3 blunt distally (pointed distally); coxa 4 moderately broad at basal part (narrow); propodi of pereopods 5 and 6 moderately short, 1.4 times as long as carpi (long, 1.7–1.9 times as long as carpi); basis of pereopod 7 broad, posterior margin convex (narrow, posterior margin slightly concave); inner ramus of uropod 1 broad, as long as outer ramus (narrow, shorter than outer ramus); and telson with deep and narrow Y-shaped excavation, without setae (deep and broad V-shaped excavation, with two pairs of setae distally). *Epimeria abyssalis* differs from *Epimeria yaquinae* in the following features (those of *Epimeria yaquinae* in parentheses): palmar margins of propodi of gnathopods 1 and 2 without projections (with pointed projections); labrum with shallow notch distally (without notch); uropod 3 slightly shorter than uropod 2 (longer than uropod 2); rami of uropod 2 broad (narrow); and telson with deep and narrow Y-shaped excavation (deep and broad V-shaped excavation).

*Epimeria abyssalis* is the deepest recorded *Epimeria* species. *Epimeria* was previously known down to 3710 m (*Epimeria glaucosa* J.L. Barnard, 1961).

#### Etymology.

Species name was derived from *abyssus* (L.) referring to its deep-water habitat.

### Key to the north Pacific species of *Epimeria*

**Table:** 

1	Rostrum short, not reaching half the length of article 1 of antenna 1; coxa 5 lacking posterodistal projection	**2**
–	Rostrum long, reaching half the length of article 1 of antenna 1; coxa 5 with posterodistal projection	**4**
2	Telson with deep and broad V-shaped excavation	**3**
–	Telson with deep and narrow Y-shaped excavation	***Epimeria abyssalis* sp. n.**
3	Eyes absent; palmar margins of gnathopods 1 and 2 with posterior projection; coxa 4 rounded distally	***Epimeria yaquinae***
–	Eyes present; palmar margins of gnathopods 1 and 2 without posterior projections; coxa 4 pointed distally	***Epimeria pelagica***
4	Eyes present	**5**
–	Eyes absent	***Epimeria subcarinata***
5	Coxa 5 projection nearly reaching epimeral plate 1	**6**
–	Coxa 5 projection not reaching epimeral plate 1	***Epimeria cora***
6	Head ventral lobe not produced,	**7**
–	Head ventral lobe produced	***Epimeria pacifica***
7	Telson 1.2 times as long as wide; uropodal peduncle longer than rami	***Epimeria morronei***
–	Telson as long as wide; uropodal peduncle shorter than rami	***Epimeria ortizi***

## Supplementary Material

XML Treatment for Epimeria

XML Treatment for Epimeria abyssalis
